# STAT3 induces G9a to exacerbate HER3 expression for the survival of epidermal growth factor receptor-tyrosine kinase inhibitors in lung cancers

**DOI:** 10.1186/s12885-019-6217-9

**Published:** 2019-10-16

**Authors:** Yi-Fang Chang, Ken-Hong Lim, Ya-Wen Chiang, Zong-Lin Sie, Jungshan Chang, Ai-Sheng Ho, Chun-Chia Cheng

**Affiliations:** 1 Division of Hematology and Oncology, Department of Internal Medicine, Mackay Memorial Hospital, Taipei, Taiwan; 2 Laboratory of Good Clinical Research Center, Department of Medical Research, Mackay Memorial Hospital, Tamsui District, New Taipei City, Taiwan; 3 Department of Medicine, Mackay Medical College, New Taipei City, Taiwan; 4 Institute of Molecular and Genomic Medicine, National Health Research Institute, Miaoli, Taiwan; 5 Graduate Institute of Medical Sciences, School of Medicine, College of Medicine, Taipei Medical University, Taipei, Taiwan; 6 Division of Gastroenterology, Cheng Hsin General Hospital, Taipei, Taiwan; 7 Radiation Biology Research Center, Institute for Radiological Research, Chang Gung University / Chang Gung Memorial Hospital at Linkou, Taoyuan, Taiwan

**Keywords:** BBI608, EGFR, G9a, HER3, Lung cancer, STAT3

## Abstract

**Background:**

HER3 mediates drug resistance against epidermal growth factor receptor (EGFR)-tyrosine kinase inhibitors (TKIs), resulting in tumor relapse in lung cancers. Previously, we demonstrated that EGFR induces HER3 overexpression, which facilitates the formation of cancer stem-like tumorspheres. However, the cellular mechanism through which EGFR regulates HER3 expression remains unclear. We hypothesized that EGFR downstream of STAT3 participates in HER3 expression because STAT3 contributes to cancer stemness and survival of EGFR-TKI resistant cancers.

**Methods:**

First, RNAseq was used to uncover potential genes involved in the formation of lung cancer HCC827-derived stem-like tumorspheres. EGFR-positive lung cancer cell lines, including HCC827, A549, and H1975, were individually treated with a panel containing 172 therapeutic agents targeting stem cell-associated genes to search for potential agents that could be applied against EGFR-positive lung cancers. In addition, gene knockdown and RNAseq were used to investigate molecular mechanisms through which STAT3 regulates tumor progression and the survival in lung cancer.

**Results:**

BBI608, a STAT3 inhibitor, was a potential therapeutic agent that reduced the cell viability of EGFR-positive lung cancer cell lines. Notably, the inhibitory effects of BBI608 were similar with those associated with YM155, an ILF3 inhibitor. Both compounds reduced G9a-mediated HER3 expression. We also demonstrated that STAT3 upregulated G9a to silence miR-145-5p, which exacerbated HER3 expression in this study.

**Conclusions:**

The present study revealed that BBI608 could eradicate EGFR-positive lung cancers and demonstrated that STAT3 enhanced the expression of HER3 through miR-145-5p repression by G9a, indicating that STAT3 is a reliable therapeutic target against EGFR-TKI-resistant lung cancers.

## Background

The overexpression and activation of the epidermal growth factor receptor (EGFR), a transmembrane receptor tyrosine kinase that belongs to the ERBB family, facilitates tumor survival, proliferation, and cancer stemness in lung cancer [1, 2]. Therefore, EGFR-tyrosine kinase inhibitors (TKIs), such as gefitinib, afatinib, and osimertinib, specifically targeting EGFR wild-type (WT), EGFR and HER2 dual targets, and EGFR T790 M, respectively, are effective therapeutic agents for the eradication of lung cancers [3]. However, resistance to EGFR-TKIs still occurs and leads to tumor recurrence [4, 5].

Various mechanisms promote EGFR-TKI resistance. In addition to KRAS and EGFR T790 mutations, the expression of oncogenes, including MET [5–7], HER2 *(ERBB2)* [8], and epidermal growth factor receptor 3 (HER3, *ERBB3*) [5, 9, 10], is associated with drug resistance against EGFR-TKIs and leads to tumor recurrence in lung cancers. For example, long-term treatment leads to complement activation of the MET-mediated signaling pathway in lung HCC827 cells and consequent overexpression of HER3 against gefitinib [5]. Antitumor therapeutics by antibodies targeting HER3 triggers a response to EGFR-TKI erlotinib in refractory non–small-cell lung cancer [11]. Constitutive overexpression of HER2 forms dimerization with HER3, leading to the downstream activation of PI3K signaling and tumor survival [12, 13]. Such results indicate that HER3 causes EGFR-TKI resistance. Therefore, it is vital to explore the molecular mechanism through which HER3 expression is regulated in EGFR-positive lung cancers.

We previously demonstrated that EGFR induces HER3 overexpression to promote the formation and survival of HCC827- and A549-derived cancer stem-like tumorspheres [14]. Because transducer and activator of transcription 3 (STAT3) contributes to cancer stemness [15, 16] and EGFR-TKI survival [17], we assume that STAT3 plays a major role in the regulation of HER3 expression. In addition, we revealed that EGFR phosphorylation participates in tumorsphere formation through the upregulation of the expression of G9a histone methyltransferase (HMT) [18]. Because YM155, an interleukin enhancer-binding factor 3 (ILF3) inhibitor [19], can block EGFR autophosphorylation to inhibit G9a-mediated stemness [18], EGFR downstream of G9a may also regulate cancer stemness and HER3 expression. Thus, the present study investigated the role of STAT3 and G9a in cancer stemness and HER3 expression.

G9a (*EHMT2*) has been reported to be an epigenetic regulator, which biochemically catalyzes the mono- and di-methylation of H3K9 (H3K9me1 and H3K9me2) in euchromatin [20], leading to gene repression [21]. Recently, G9a has been demonstrated to interact with several transcriptional factors, including GATA3 and ZEB2 [22], STAT3 [16], and MYC [23], leading to the repression of gene transcription, while enhancing tumor survival. G9a is a potential mediator that silences tumor suppressors based on interacting partners. Particularly, G9a was reported to reduce the expression levels of microRNAs, such as miR-200c, under the mediation of STAT3-G9a, which causes the astrocyte leptin receptor to exacerbate tumor progression in breast cancer [16]. In addition, G9a interacts with MYC, which drives transcriptional repression and tumorigenesis [23]. To the best of our knowledge, STAT3 also induces MYC expression, and both transcriptional factors have been reported to participate in tumor stemness [24–27]. STAT3 was demonstrated as a target against EGFR-TKI resistance [21]. Therefore, we assumed that EGFR promotes HER3 overexpression through the STAT3-mediated activation of G9a to repress the expression of HER3-targeted microRNAs, because we identified that STAT3, an EGFR downstream phosphorylated target, participates in tumorsphere formation and survival in EGFR-positive colorectal cancer cell lines [15].

To validate the aforementioned assumptions, we used RNAseq to explore differential genes participating in the formation of HCC827-derived stem-like tumorspheres and in the knockdown of A549shSTAT3 and A549shG9a lung cancer cell lines compared with A549shLuc controls. The RNAseq-based gene profiling by treatment of BBI608, which selected by a screened panel targeting to stem-associated genes, and the inhibitory effects of BBI608 were compared with those of YM155, which particularly inhibited the formation of tumorspheres. BBI608, a STAT3 inhibitor, reduced not only the viability of EGFR-positive lung cancer cell lines but also the expression of G9a and HER3. These results were consistent with findings obtained following treatment with YM155, an ILF3 inhibitor. In addition, we demonstrated that STAT3 upregulated G9a expression, which significantly silenced miR-145-5p and exacerbated HER3 expression. The present study revealed the role of the transcriptional repression of miRNAs, such as miR-145-5p, by the STAT3-G9a axis in EGFR-positive lung cancers, which exacerbated HER3 expression and led to EGFR-TKI resistance.

## Methods

### Cell culture and tumorsphere formation

HCC827 (CRL-2868), A549 (CCL-185), H1975 (CRL-5908), and H520 (HTB-182) lung cancer cell lines were purchased from the American Type Culture Collection (ATCC, Manassas, VA, USA). The cell lines were free from *Mycoplasma*. HCC827, H1975, and H520 cell lines were cultured in RPMI-1640 medium with 10% *fetal bovine serum (FBS) and 1%* penicillin–streptomycin. A549 was cultured in Dulbecco’s modified Eagle medium with the same additives. The cell lines were reauthenticated through short tandem repeat profiling (Applied Biosystems, Massachusetts, USA): HCC827 on May 8, 2015; A549 on June 4, 2014; H1975 on May 23, 2019; H520 on December 13, 2016. For tumorsphere formation, cells were cultured in low-attached six-well plates with serum-free medium containing B27 (Invitrogen, Waltham, MA), 20 ng/mL of EGF (Sigma, Missouri, TX), 20 ng/mL of fibroblast growth factor (bFGF, Sigma), 5 μg/mL of bovine insulin (Sigma), and 4 μg/mL of heparin (Sigma) for at least a 7-day incubation period. The sizes of tumorspheres were examined under an inverted microscope (Axio Observer 3, ZEISS, Oberkochen, Germany). All cells were incubated at 37 °C and 5% CO_2_.

### Animals

Male NOD/SCID mice were purchased from BioLASCO Taiwan Co., Ltd., Taiwan. Five-week-old mice were maintained under a 12-h light/dark cycle at 22 °C. Animal studies were approved by the Institutional Ethical Review Committee at Mackay Memorial Hospital, Taiwan, and were performed according to NIH guidelines on the care and welfare of laboratory animals. Tumor xenografts were established by injecting 2 × 10^6^ of A549shLuc (*n* = 4) or A549shSTAT3 (*n* = 4) into the subcutaneous legs of 5-week-old mice. For tumor growth inhibition, 10 mg/kg of BBI608 was injected via tail vein in A549-derived tumor xenografts (*n* = 3 for each group). Tumors were externally measured using a digital caliper, and tumor volumes were calculated using the following formula: 0.52 × width^2^ × length, where the smaller tumor diameter represented the width. Animals were sacrificed using carbon dioxide inhalation.

### RNAseq, small RNAseq profiling, and bioinformatics analysis

RNAseq was performed to determine differentially expressed mRNAs in (1) HCC827-derived tumorspheres compared with parental HCC827 cells, (2) A549 cells treated with 1 μg/mL of BBI608 and 1 μg/mL of YM155, and (3) A549shSTAT3 and A549shG9a compared with A549shLuc (luciferase). A HiSeq 4000 with paired-end 150-bp sequencing was used for experiments. Genes upregulated with a > 2-fold change (log2) with a *p* value of < 0.05 in HCC827-derived tumorspheres were selected for bioinformatics analyses by using NetworkAnalyst (http://www.networkanalyst.ca/) [28], and pathway activations were selected and matched based on the KEGG database. Genes downregulated with a less than − 1-fold change (log2) with a *p* value of < 0.05 in (1) BBI608- and YM155-treated A549 cells compared with parental A549 cells and (2) A549shSTAT3 and A549shG9a compared with A549shLuc cells were compared using List Operations (http://www.molbiotools.com/listoperations.html) to determine common genes that were differentially expressed. In addition, differentially expressed genes were analyzed using NetworkAnalyst to determine major signaling pathways involved and key genes. Differentially expressed microRNAs were investigated using small RNA digitalization analysis through sequencing by synthesis (Illumina, San Diego, California, USA). The expression levels of known and unique miRNAs in each sample were statistically analyzed and normalized using transcripts per million clean tags (TPMs) [29]. Common differential miRNAs in A549shILF3 and A549shG9a identified using List Operations were compared with predictable HER3-binding miRNAs selected by TargetScan (http://www.targetscan.org/vert_72/) based on conserved sites for broadly conserved miRNA families among vertebrates [30].

### Quantitative PCR

The mRNA extraction and cDNA preparation were performed as described previously [18]. Quantitative PCR (Applied Biosystems, California, USA) was performed using the SYBR Green system (Applied Biosystems, California, USA) according to manufacturer’s instructions. Primers used for PCR were as follows: *ERBB3* (HER3): forward, 5′-GCCAATGAGTTCACCAGGAT-3′ and reverse, 5′-ACGTGGCCGATTAAGTGTTC-3′. *GAPDH*: forward, 5′- GAGTCAACGGATTTGGTCGT-3′ and reverse, 5′- TTGATTTTGGAGGGATCTCG-3′.

### Gene knockdown and overexpression

Gene knockdown was performed using a short-hairpin RNA (shRNA)-expression lentivirus system that contained the specific shRNA (target sequence of *STAT3*, *ILF3*, and *EHMT2* (G9a): GCACAATCTACGAAGAATCAA, GCCATGTGATGGCAAAGCATT, and GCTCCAGGAATTTAACAAGAT for shG9a#1 / CGAGAGAGTTCATGGCTCTTT for shG9a#2, respectively) in the pLKO.1-puro vector generated in a 293 T cell line. For G9a overexpression in A549 cells, pLenti6-MK1-EHMT2-V5 based on lentiviral system (Addegene, Massachusetts, USA) was purchased and used. The procedure followed was the same as that in our previous study [18].

### Western blot analysis

Western blot analysis was performed as described previously [18]. Specific antibodies against ILF3, G9a, di-mH3K9, STAT3, pSTAT3, HER3, and GAPDH were purchased from Cell Signaling (Danvers, Massachusetts, USA). Image J software was used to calculate the G9a, di-mH3K9, HER3 ratio divided by GAPDH.

### Cell viability

The Alarmar Blue assay was performed according to manufacturer’s instructions to determine cell viability. To find therapeutic agents that can be applied against EGFR-positive lung cancers, a panel containing 172 compounds targeting stemness (MCE, Monmouth Junction, NJ, USA) was added to HCC827, A549, H1975, and H520 cell lines separately at doses of 1 μM and incubated for 48 h. To examine cell viability, cells were treated using afatinib, BBI608, or YM155 for 48 h.

### Cell migration

Transwell migration assay (8 μm) was used to detect A549 cell migration capacity. In brief, 5 × 10^4^ cells were placed in the upper layer of a cell culture insert with 200 μL of serum free DMEM medium. To test cell migration of A549-derived tumorspheres, serum-free DEME medium containing B27 (Invitrogen, Waltham, MA), 20 ng/mL of EGF (Sigma, Missouri, TX), 20 ng/mL of fibroblast growth factor (bFGF, Sigma), 5 μg/mL of bovine insulin (Sigma), and 4 μg/mL of heparin (Sigma) was used. Then, each 750 μL of DMEM medium with 10% FBS and test agents, including 1 μg/mL BBI608, 1, 5, 10 μg/mL UNC0642, was loaded into the below 24-well culture plate. Cells were incubated at 37 °C and 5% CO_2_ for 16 h. The membrane inserts were fixed in 3.7% formaldehyde for 5 min and consequently incubated in 100% methanol for 20 min at room temperature. After 0.5% crystal violet in 2% ethanol to stain the membrane inserts for 15 min at room temperature, non-migrated cells on the upper membrane were scraped with cotton swabs. PBS wash for twice was necessary between operations. The cells migrated through the membrane were imaged and counted using an inverted microscope (Axio Observer 3, ZEISS, Oberkochen, Germany).

### Measurement of miR-145-5p

A TaqMan advanced miRNA assay (Applied Biosystems, California, USA) was used to detect the expression of miR-145-5p in (1) lung cancer cell lines, including HCC827, A549, and H1975, (2) A549 cells treated with 1 μg/mL of BBI680 for 48 h, and (3) A549shG9a with or without 20 ng/mL of EGF treatment in comparison with A549shLuc. The detection of miR-145-5p was according to manufacturer’s instructions.

### Statistical analysis

Statistical analyses were performed using GraphPad Prism v5.01 (GraphPad Software, Inc., California, USA). All analytical data with more than two groups were evaluated using analysis of variance, followed by post hoc analysis with Bonferroni’s test. Student’s t test was used to compare two groups. In addition, *p* < 0.05 was considered to indicate a statistically significant difference.

## Results

### HER3 overexpressed in HCC827-derived stem-like tumorspheres

**Fig. 1:**
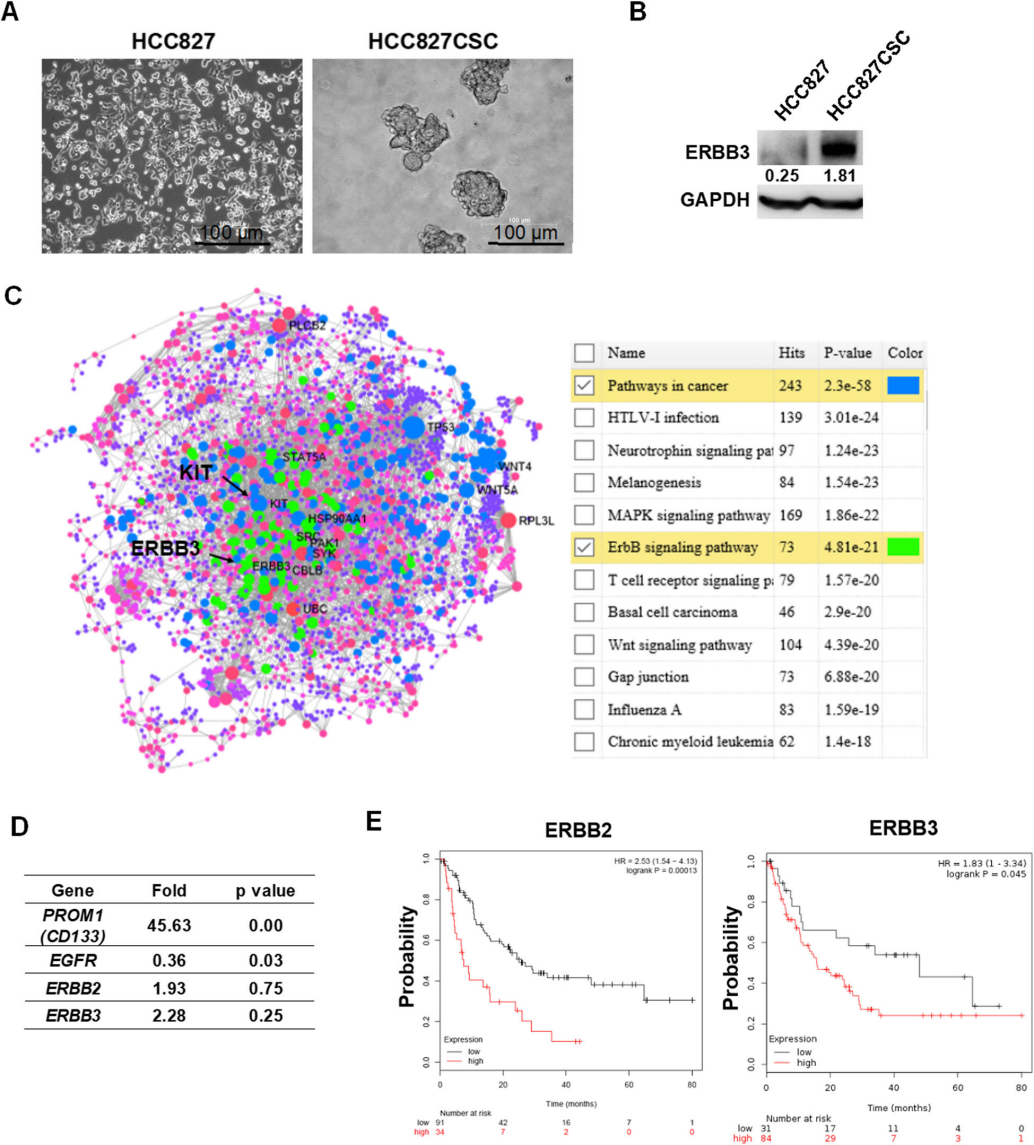
HER3 overexpressed in lung cancer HCC827-derived tumorspheres. **a** HCC827-derived tumorspheres were cultured in serum-free RPMI-1640 medium with the addition of EGF, bFGF, insulin, and heparin and incubated for 7 days. Tumorspheres were approximately 100 μm in diameter, measured using an inverted microscope. Bar scale, 100 μm. **b** HER3 (*ERBB3*) was detected through Western blot, which revealed high expression in HCC827-derived tumorspheres. **c** RNAseq was used to search and validate differential genes involved in the formation of tumorspheres. The results validated the overexpression of *KIT* and *ERBB3* (a detailed gene list shown in Additional file 1: Table S1), **d** and *ERBB2* and CD133 (*PROM1*), a marker for cancer stemness, also increased. **e** To validate that the expression of *ERBB2* and *ERBB3* were associated with the patient survival rate, a Kaplan Meier plotter was used (http://kmplot.com/analysis/). Results revealed that higher *ERBB2* and *ERBB3* individually reduced the probability of survival in patients with lung adenocarcinoma (*p* = 0.00013 for *ERBB2* and *p* = 0.013 for *ERBB3*)

To validate that HER3 was overexpressed in lung cancer stem cells, we cultured lung cancer HCC827 cells in the serum-free medium described in Methods and Materials to develop an artificial stem-like cancer model (Fig. 1a), which expressed a high level of CD133, as demonstrated previously [18]. HER3 was overexpressed in HCC827-derived tumorspheres analyzed through Western blot (Fig. 1b). Subsequently, RNAseq was used to find key genes involved in tumorsphere formation. The entire gene expression dynamics are presented in Additional file 1: Table S1. Based on NetworkAnalyst results and the matching of data based on KEGG datasets associated with pathways in cancers, *KIT* was identified, which is also a stem cell factor (Fig. 1c). In addition, KEGG searches linked to the ERBB signaling pathway validated HER3 (*ERBB3*) overexpression in tumorspheres (Fig. 1c). In addition, increased expression of genes belonging to the ERBB family, including *EGFR*, was detected by RNAseq, whereas the expression of CD133 (*PROM1*), a stem cell marker, significantly increased in HCC827 stem-like tumorspheres (Fig. 1d). To ensure that selected genes, namely *ERBB2* and *ERBB3*, were oncogenes associated with lung cancer, a Kaplan–Meier plotter [31] was then used. According to results, the overexpression of *ERBB2* and *ERBB3* reduced the probability of survival of clinical patients with lung adenocarcinoma (Fig. 1e).

### BBI608, a STAT3 inhibitor, significantly reduced EGFR-positive lung cancers to against EGFR-TKI-resistance

**Fig. 2:**
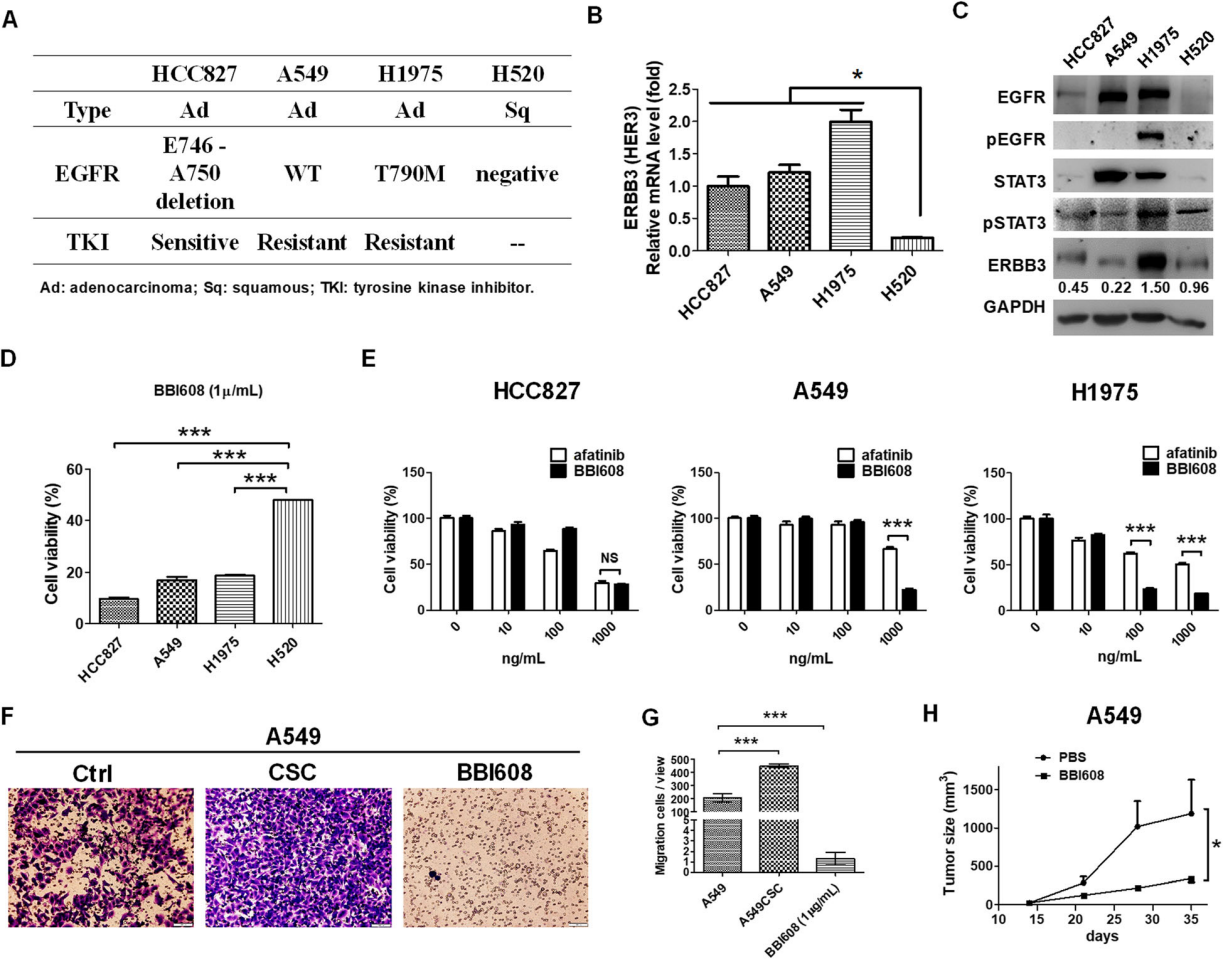
BBI608 significantly inhibited cell viability in EGFR-positive lung cancers, including EGFR E746-A750 HCC827, wild-type (WT) A549, and T790 M H1975. **a** EGFR-positive HCC827, A549, and H1975 and EGFR-negative H520 lung cancers were selected for use in identifying potential therapeutic agents. HCC827 with EGFR E746-A750 mutation triggers TKIs, whereas A549 and H1975 bearing KRAS and T790 M mutations, respectively, are resistant to TKIs. **b** Results from qPCR revealed that *ERBB3* was expressed in HCC27, A549, and H1975 at higher levels than in H520 cells. In particular, H1975 showed the highest *ERBB3* expression level. **c** The results of Western blot analyses demonstrated and validated that H1975 with EGFR autophosphorylation presented higher phosphorylation of STAT3 and expression of HER3 (*ERBB3*). **d** To identify potential therapeutic agents against EGFR-positive lung cancers, a panel kit containing 172 compounds was used and HCC827, A549, H1975, and H520 cells were added separately and incubated for 48 h. The detailed cell viability measurements are illustrated in Additional file 9: Figure S1, which revealed that BBI608 significantly reduced the viability of HCC827, A549, and H975 cells as opposed to the viability of H520 cells. **e** BBI608 significantly reduced the viability of HCC827 cells, which is similar to the results observed under afatinib, an EGFR-TKI. However, BBI608 reduced the viability of A549 and H1975 cells compared with the viability of these cells under afatinib, indicating that BBI608 overcame EGFR-TKI resistance associated with KRAS and T790 M mutations. **f** and **g** A549-derived cancer stem-like cells significantly increased cell migration, but A549 treated with BBI608 reduced cell migration. **h** Moreover, BBI608 significantly reduced tumor growth in A549-transplated xenografts in vivo. *p < 0.05, ***p < 0.001, NS, nonsignificant. Scale bar, 100 μm

To find potential therapeutic agents against EGFR-positive lung cancers, particularly for reducing drug resistance associated with HER3, four lung cancer cell lines, namely EGFR-positive HCC827, A549, and H1975 cells and EGFR-negative H520 cells, were selected and investigated in the present study. The characteristics of the selected cell lines are illustrated in Fig. 2a. HCC827 is an EGFR E746-A750 deletion that is sensitive to EGFR-TKIs. A549 is an EGFR WT but with a KRAS mutation that is resistant to EGFR-TKIs. H1975 with an EGFR T790 M mutation also resists EGFR-TKI due to EGFR autophosphorylation. We observed that the expression of HER3 in EGFR-positive cell lines was higher than that in EGFR-negative H520 cells analyzed using qPCR (Fig. 2b). The results of Western blot were consistent and demonstrated high HER3 expression in H1975 cells that exhibited the simultaneous autophosphorylation of EGFR and increased phosphorylation of STAT3 than in other cell lines (Fig. 2c). In addition, a panel containing 172 therapeutic agents targeting genes associated with various stem cell pathways was used, and the viability of each cell line was measured after incubation with 1 μg/mL of therapeutic agents for 48 h. The results are presented in Additional file 9: Figure S1. BBI608, a stemness inhibitor targeting the STAT3 pathway, significantly reduced selected EGFR-positive cell lines, with a cell viability less than 40%. In addition, BIO targeting of CDK1 and GSK-3 reduced HCC827 and H1975 cell viability. Sanguinarine targeting of ERK particularly reduced A549 cell viability. The compound static targeting of STAT3 and halofuginone targeting of TGFβ/smad reduced cell viability both in A549 and H1975 cells. TG101209 targeting of JAKs specifically reduced cell viability against H1975 cells. The decrease in the viability of HCC827, A549, and H1975 cells following treatment with BBI608 was validated, and the viability of cell lines was lower than that of H520 cells (Fig. 2d). In addition, there was no significant difference between the decrease in cell viability caused by afatinib and BBI608 in HCC827 cells; however, BBI608 caused a substantial reduction in the viability of both A549 and H1975 cells (Fig. 2e), indicating that STAT3, the BBI608 target gene, mediated EGFR-TKI resistance. We also found that cell migration capacity was increased in A549 cells-derived cancer stem-like cells in vitro, which was reduced in 1 μg/mL of BBI608 treatment (Fig. 2f and g). We found BBI608 was able to induce cell apoptosis in vitro (Additional file 12: Figure S4). BBI608 also inhibited tumor growth in A549-derived tumor xenografts in vivo (Fig. 2h). The results revealed that BBI608 was a potential therapeutic agent against lung cancer.

### STAT3 contributes to G9a and HER3 expression and influences cell survival in HCC827-derived tumorspheres

**Fig. 3:**
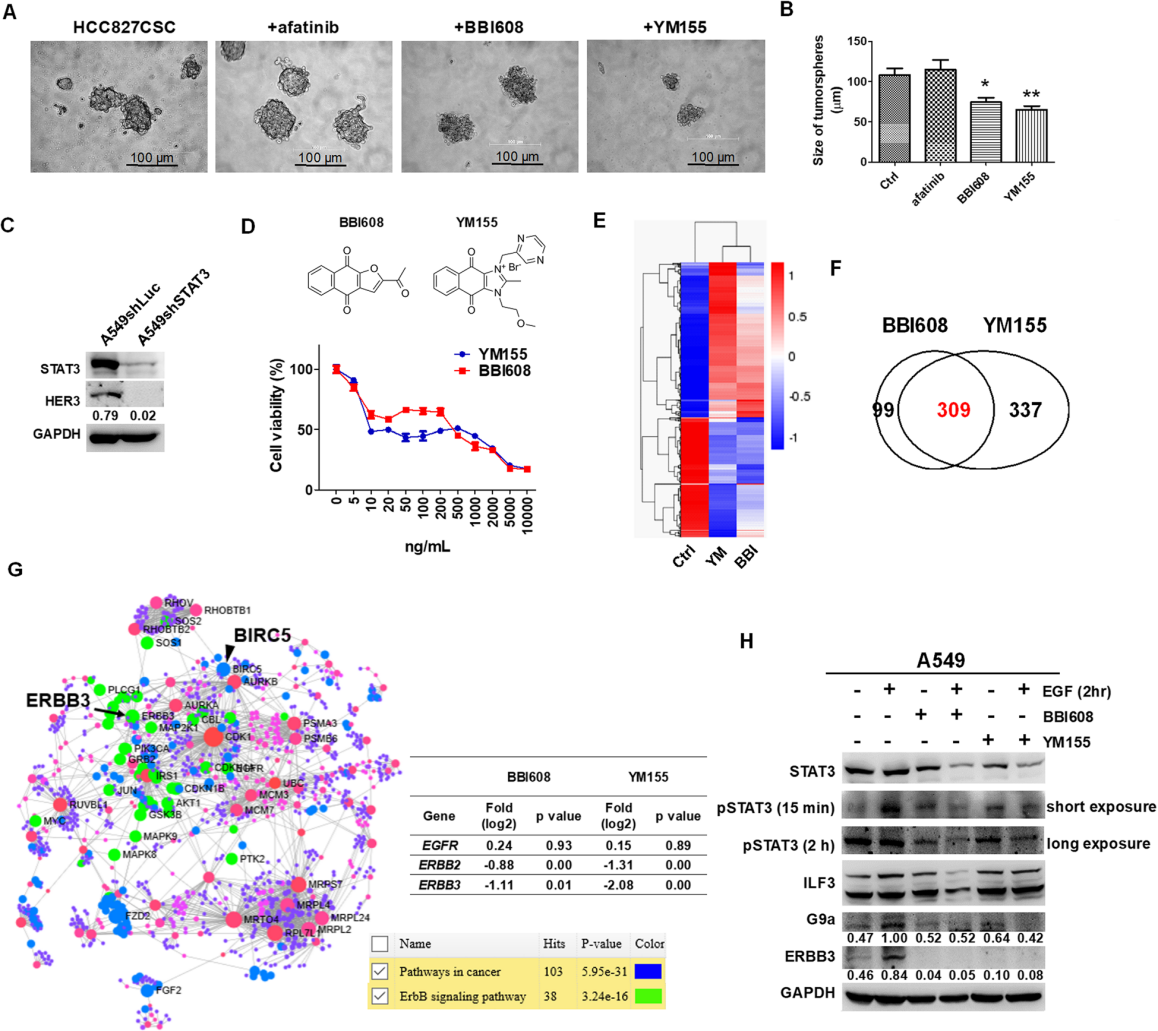
BBI608 and YM155 inhibited STAT3 phosphorylation and G9a and ERBB3 expression in EGFR-positive lung cancers. **a** To validate the inhibitory capacity of BBI608 and YM155 against the formation of cancer stemness, HCC827-derived stem-like cancer tumorspheres (HCC827CSC) were treated separately with 1 μg/mL of afatinib, BBI608, and YM155 over a 7-day incubation. We demonstrated that BBI608 and YM155 could reduce the formation of HCC827-derived tumorspheres compared with afatinib as observed under an inverted microscope. **b** The tumorsphere sizes of HCC827CSC were measured and compared. Results showed that BBI608 and YM155 significantly reduced HCC827CSC formation, whereas afatinib exhibited no effect at a concentration of 1 μg/mL. **c** There were lower levels of HER3 in A549shSTAT3 compared with A549shLuc, indicating that STAT3 facilitated HER3 expression. **d** In addition, BBI608 and YM155 caused a similar reduction in the viability of A549 cells in a dose-dependent manner. **e** Gene cluster profiling from the RNAseq analysis revealed a similar pattern between BBI608- and YM155-treated A549 cells (Additional file 2: Table S2 and Additional file 3: Table S3). **f** There were 309 common differentially expressed genes between BBI608- and YM155-treated A549 cells (Additional file 6: Table S6), **g** which were consequently analyzed by NetworkAnalyst to identify key potential genes mediated by BBI608 and YM155. Results revealed and validated reductions in *ERBB2* and *ERBB3* expression, whereas *BIRC5* is an inhibitory target of YM155. **h** In addition, BBI608 and YM155 substantially reduced endogenous and EGF-mediated STAT3 phosphorylation, resulting in G9a and HER3 (*ERBB3*) reductions. *p < 0.05, **p < 0.01. Scale bar, 100 μm

To validate that STAT3 was a therapeutic target against lung cancer stem cells, HCC827-derived tumorspheres were incubated with 1 μg/mL of afatinib, BBI608, and YM155, and the diameters of tumorspheres were measured. In our previous studies, YM155, an ILF3 inhibitor, was demonstrated to be an efficient agent against the formation of cancer stem-like tumorspheres and HER3 expression [14, 18]. As expected, BBI608 and YM155 significantly reduced tumorsphere formation, whereas afatinib had no effect (Fig. 3a and b). Subsequently, STAT3 was knocked down in A549 cells, and results revealed a reduction in HER3 expression in A549shSTAT3 cells compared with A549shLuc cells (Fig. 3c). Because both BBI608 and YM155 inhibited tumorsphere formation in vitro, the capacities of the two therapeutic agents to alleviate inhibition were measured against A549 cells. Results revealed that BBI60 and YM155 had similar inhibitory effects in the 0–10 μg/mL concentration range (Fig. 3d), which implied that the two compounds had a similar inhibitory target. The transcriptomic profiling analysis based on RNAseq revealed consistent gene expressional clusters in BBI608- and YM155-treated A549 cells (Fig. 3e). Differentially expressed genes are listed in Additional file 2: Table S2 for BBI608-treated A549 cells and in Additional file 3: Table S3 for YM155-treated A549 cells. A total of 309 downregulated genes were common between BBI608 and YM155 treatments (Fig. 3f and Additional file 6: Table S6); these genes were associated with pathway with cancer and the ERBB signaling pathway, which included *ERBB3* (HER3) and *BIRC5* (Survivin) (Fig. 3g), whereas *BIRC5* was previously identified as an inhibitory gene of YM155 [32]. In addition, *ERBB2* decreased following treatment with the two inhibitors (Fig. 3g). These results suggest that STAT3 was a target of YM155. Therefore, we investigated STAT3 phosphorylation and G9a expression in BBI608 and YM155 treatments with or without EGF co-treatment in A549 cells, whereas G9a was demonstrated as a downregulated target of YM155 [18]. Both BBI608 and YM155 reduced the endogenous and EGF-induced phosphorylation of STAT3, resulting in decreases in G9a and HER3 expression (Fig. 3h).

### G9a mediated STAT3-regulated HER3 expression in EGFR-positive lung cancer

**Fig. 4:**
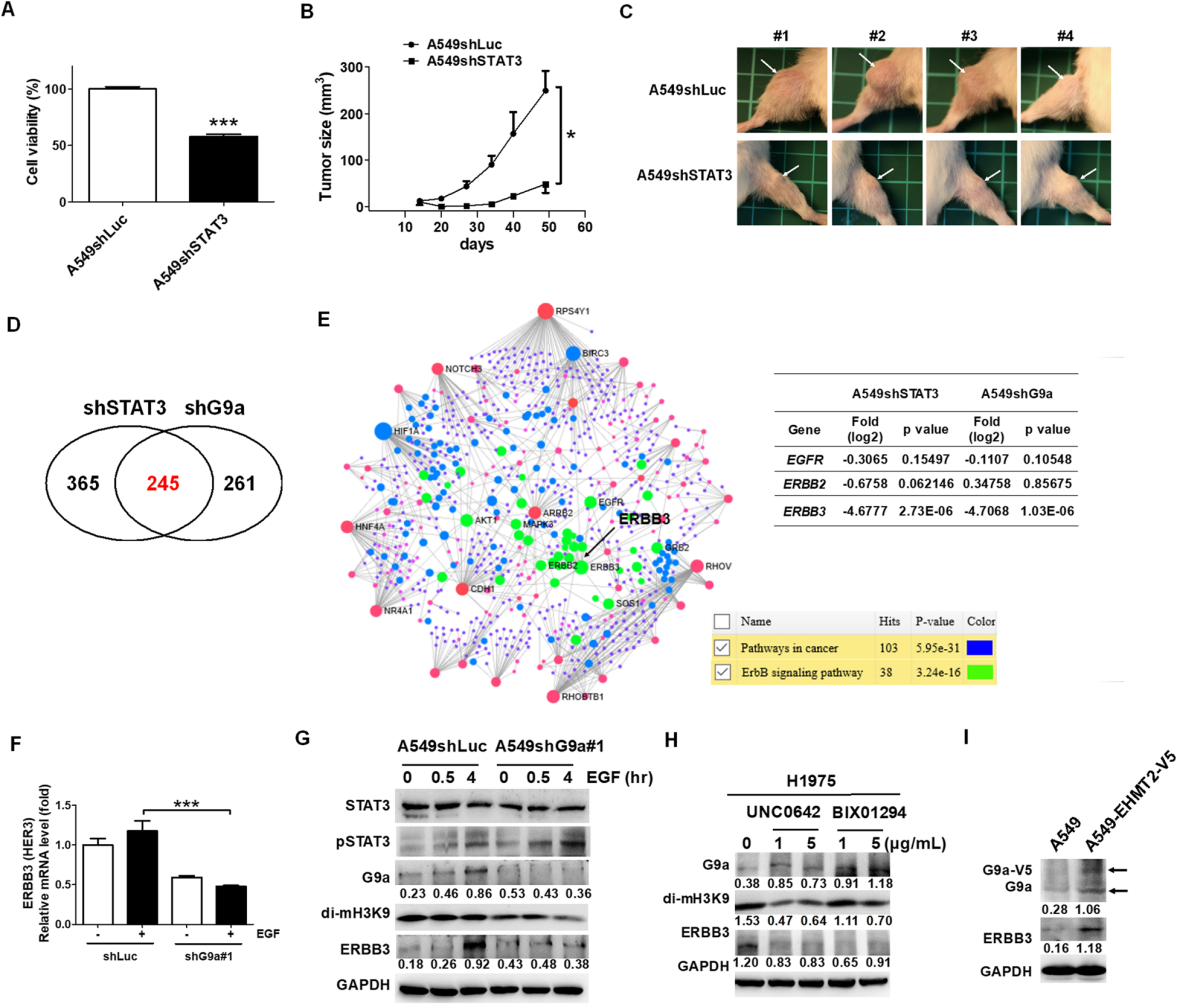
STAT3-mediated G9a facilitated the expression of ERBB3. **a** Knockdown of STAT3 in A549 cells reduced cell viability in vitro, and **b** and **c** in vivo in a tumor xenograft model. Tumors are indicated by arrows. **d** There were 245 common genes between RNAseq data from A549shSTAT3 (Additional file 4: Table S4) and A549shG9a (Additional file 5: Table S5), **e** including *ERBB3* after NetworkAnalyst analysis. In addition, *ERBB2* expression decreased only in A549shSTAT3. The data revealed that G9a led to ERBB3 expression in A549 cells. **f** To validate the assumption, *ERBB3* was detected by qPCR in EGF-treated A549shG9a cells compared with A549shLuc cells. The results indicated that *ERBB3* decreased in A549shG9a cells with or without EGF treatment. **g** The results from Western blot analyses revealed that EGF induced STAT3 phosphorylation resulting in G9a and ERBB3 overexpression. Knockdown of G9a reduced the EGF-mediated ERBB3 expression without influencing STAT3 phosphorylation in A549 cells, demonstrating that G9a facilitated ERBB3 expression. **h** In addition, G9a inhibitors, UNC0642 and BIX01294, significantly blocked G9a-mediated di-mH3K9 activity and reduced ERBB3 expression in EGFR-autophosphorylated lung cancer H1975 cells. **i** Meanwhile, overexpression of G9a increased ERBB3 expression in A549 cells. *p < 0.05, ***p < 0.001

Because the expression of both G9a and HER3 decreased following treatment with BBI608 and YM155, we investigated whether G9a regulated the expression of HER3 in lung cancer. First, we validated that the knockdown of STAT3 reduced the viability of lung cancer cells significantly (Fig. 4a), which is consistent with the results of our previous study that demonstrated that STAT3 facilitated the survival of colorectal cancer stem-like cells [15]. In addition, there was a significant reduction of tumor growth in vivo in the A549shSTAT3 cell line compared with the A549shLuc cell line based on a tumor xenograft model (Fig. 4b and c). However, knockdown of G9a did not affect in vitro cell viability in A549 cells and in vivo A549-induced tumor xenografts (Additional file 10: Figure S2). The transcriptomic profiles of A549shSTAT3 and A549shG9a cell lines analyzed using RNAseq were compared with A549shLuc (Additional file 4: Table S4 for A549shSTAT3 and Additional file 5: Table S5 for A549shG9a). There were 245 commonly downregulated genes between A549shSTAT3 and A549shG9a cell lines (Fig. 4d and Additional file 6: Table S6), including *ERBB3*, which was observed in the NetworkAnalyst analysis results (Fig. 4e). However, *ERBB2* expression decreased only in the A549shSTAT3 cell line. In addition, there were 55 common genes between 309 and 245 genes from the BBI608/YM155 and the shSTAT3/shG9a axis, respectively (Additional file 11: Figure S3 and Additional file 6: Table S6). The results indicated the suppression of the ERBB signaling pathway, which particularly inhibited HER3 expression. To further ensure that G9a was capable of inducing HER3 expression, qPCR and Western blot analyses were performed to investigate HER3 expression in EGF-treated A549shG9a cell lines and H1975 cell lines treated with G9a inhibitors, including UNC0642 and BIX01294. Results showed a considerable decrease in HER3 expression in EGF-treated A549shG9a cell lines compared with EGF-treated A549shLuc cell lines (Fig. 4f). In addition, EGF-mediated STAT3 phosphorylation increased HER3 expression in a time-dependent manner, which was inhibited in A549shG9a cell lines without influencing EGF-mediated STAT3 phosphorylation (Fig. 4g). Moreover, UNC0642 and BIX01294 inhibited the expression of HER3 in a dose-dependent manner, whereas H3K9 dimethylation was a target of G9a, which was also inhibited by UNC0642 and BIX01294 (Fig. 4h). Overexpression of G9a led to increase of HER3 in A549 cells (Fig. 4i), suggesting that G9a contributed to the overexpression of HER3 in EGFR-positive lung cancers.

### G9a repressed miR-145-5p and exacerbated HER3 expression

**Fig. 5:**
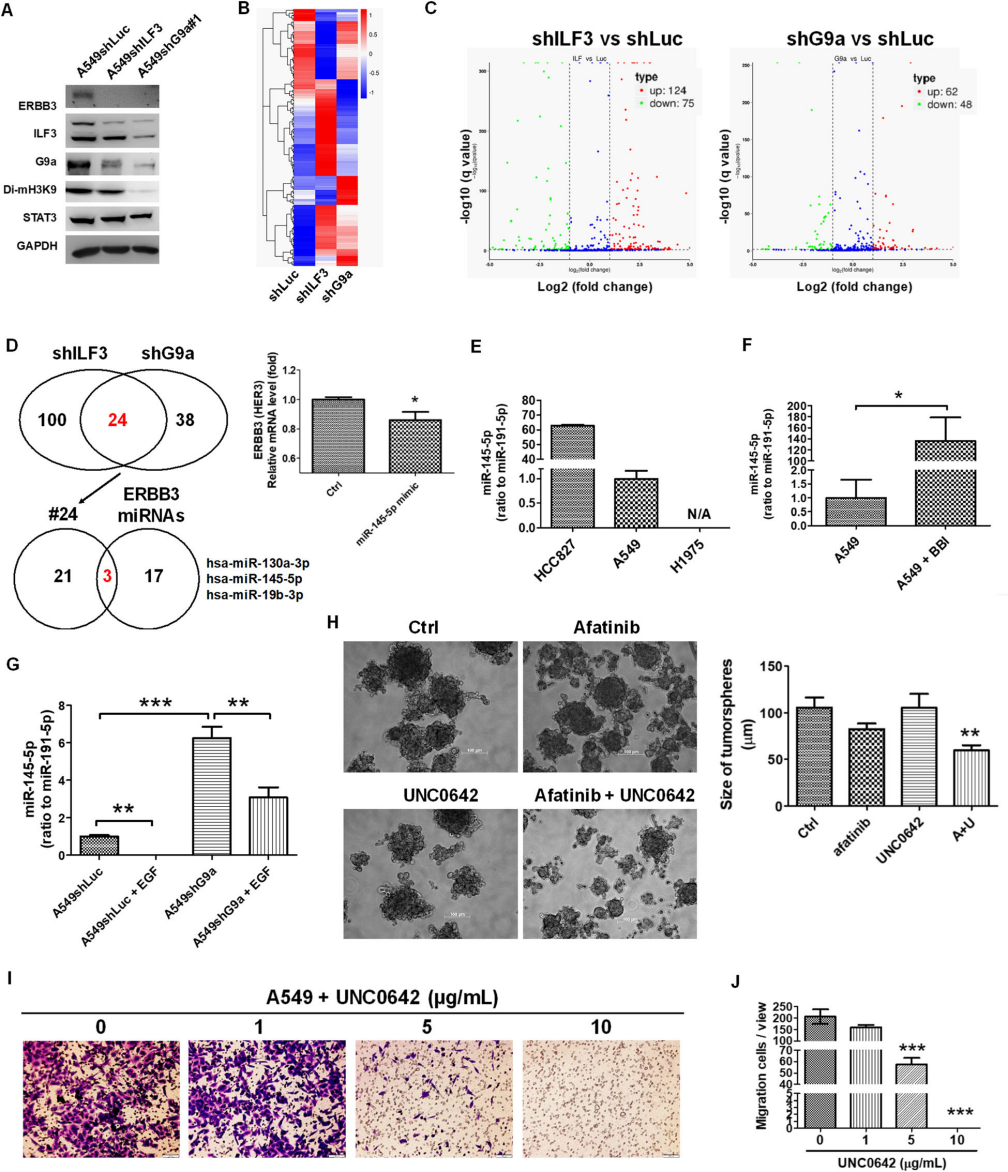
G9a induced *ERBB3* expression via the repression of miR-145-5p. **a** Western blot analysis revealed that the knockdown of ILF3 and G9a led to a reduction in HER3 (*ERBB3*) expression in A549 cells. There was a reduction of G9a in A549ILF3 cells, whereas demethylation of H3K9 presented G9a activity. **b** The gene cluster profiles in A549shILF3 and A549shG9a were listed and compared following small RNAseq analysis, **c** which revealed that 124 and 75 miRNAs were upregulated and downregulated, respectively, in A549shILF3 (Additional file 7: Table S7), and 62 and 48 miRNAs were upregulated and downregulated, respectively, in A549shG9a cells compared with A549shLuc cells (Additional file 8: Table S8). **d** There were 24 miRNAs with increased expression that were common between A549ILF3 and A549shG9a, and 3 potential miRNAs involved in HER3 expression, which were compared with the HER3-targeted miRNAs from TargetScan, including miR-130a-3p, miR-145-5p, and miR-19-3p. In addition, miRNA-145-5p transfection into A549 cells reduced HER3 expression detected by qPCR. **e** The expression of miRNA-145-5p in HCC827 cells was higher than in A549 and H1975 cells. **f** BBI608 (1 μg/mL) treatment significantly increased miR-145-5p expression in A549 cells. **g** In addition, the miRNA-145-5p level significantly increased in A549shG9a compared with in A549shLuc cells, which reduced in EGF treatment. **h** Therefore, co-treatment of afatinib with UNC0642 significantly reduced HCC827-derived tumorsphere formation based on tumorsphere diameter. **i** and **j** Therefore, targeting G9a by UNC0642 also reduced cell migration in A549 cells in a dose-dependent manner. *p < 0.05, **p < 0.01, ***p < 0.001. Scale bar, 100 μm

To investigate the potential mechanism through which G9a regulated the expression of HER3, we investigated whether microRNAs were regulated and inhibited by G9a, because G9a is an HMT and has a gene silencing function. A549shILF3 and A549shG9a cell lines with a low HER3 expression level (Fig. 5a) were selected for application in investigating miRNA expression profiles by using small RNAseq. We observed that the knockdown of ILF3 reduced G9a expression levels (Fig. 5a). The gene cluster profiles of A549shILF3 and A549shG9a cell lines are illustrated in Fig. 5b, which indicated there were 124 upregulated and 75 downregulated miRNAs in the A549shILF3 cell line (Additional file 7: Table S7), and 62 and 48 miRNAs were upregulated and downregulated, respectively, in the A549shG9a cell line compared with the A549shLuc cell line (Additional file 8: Table S8) (Fig. 5c). Among increased miRNAs, there were 24 miRNAs commonly expressed between A549ILF3 and A549shG9a cell lines, which were further compared with HER3-targeted miRNAs predicted using TargetScan. We identified three miRNAs that were also predicted as HER3-targeted miRNAs, namely miR-130a-3p, miR-145-5p, and miR-19-3p (Fig. 5d). A study revealed that miR-145 is capable of targeting and inhibiting HER3 in breast cancer [33]. We found 4 genes regulated by STAT3-G9a-miR-145-5p through comparing the 55 common gene (Additional file 11: Figure S3A) with miR-145-5p-targeted gene from TargetScan, including *ERBB3* (HER3), *PLA2GA4*, *MTUS1*, *TSKU* (Additional file 11: Figure S3C). Therefore, miRNA-145-5p was transfected into A549 cells, which resulted in a decrease in the HER3 expression detected by qPCR analysis (Fig. 5d). In addition, miRNA-145-5p was expressed highly in the HCC827 cell line compared with the expression level in A549 and H1975 cell lines (Fig. 5e). miRNA-145-5p expression was negatively correlated with the expression of HER3, as illustrated in Fig. 2b. In addition, BBI608 significantly increased miR-145-5p expression (Fig. 5f). miRNA-145-5p expression levels increased significantly in A549shG9a cells compared with in A549shLuc cells, which reduced following treatment with EGF (Fig. 5g). The results demonstrated that STAT3-G9a increased HER3 expression through the repression of miR-145-5p. We then demonstrated that co-treatment of afatinib with UNC0642 significantly reduced the formation of HCC827-derived tumorspheres measured based on tumorsphere diameters (Fig. 5h), which is consistent with the findings following treatment with BBI608 and YM155 (Fig. 3a and b). In addition, UNC0642 targeting G9a reduced cell migration of A549 cells in a dose-dependent manner (Fig. 5i and j) and induced A549 cell apoptosis (Additional file 12: Figure S4).

## Discussion

**Fig. 6:**
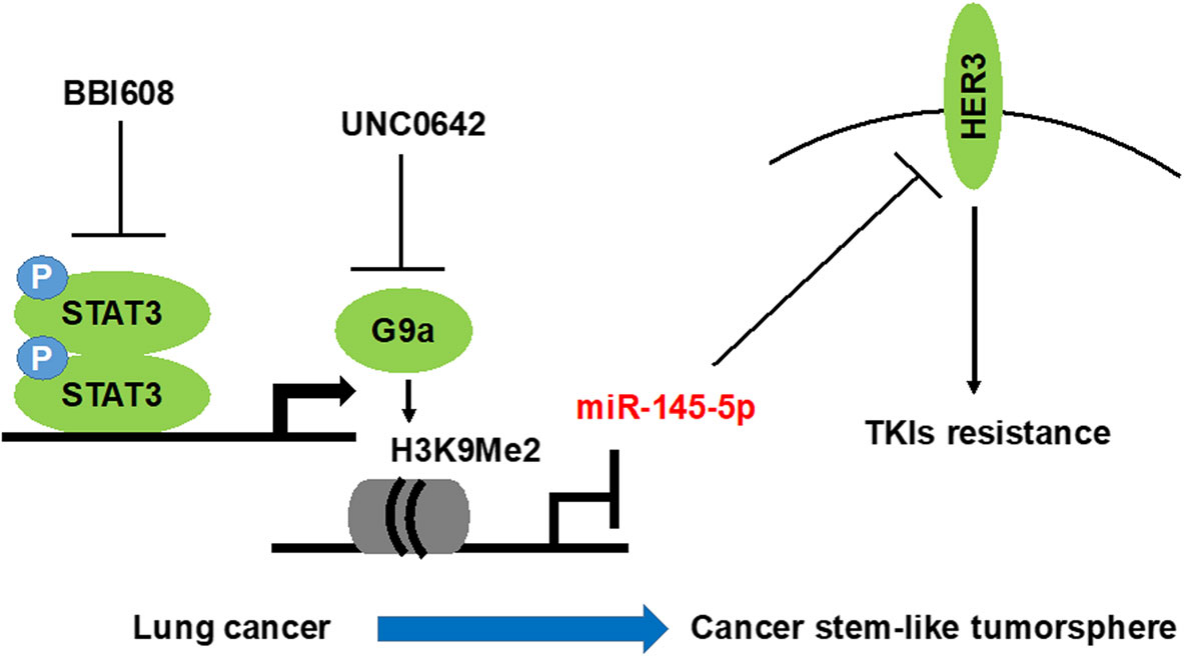
A proposed model illustrating the STAT3–G9a–miR-145-5p axis as a therapeutic target that regulates HER3 expression governing TKIs resistance in lung cancer-derived stem like-tumorspheres

The present study demonstrated that STAT3 promotes HER3 expression through the activation of G9a, which, in turn, represses HER3-targeted miR-145-5p expression in EGFR-positive lung cancers (Fig. 6). We identified a potential therapeutic agent, BBI608, which targeted STAT3, and significantly and effectively reduced cell proliferation and tumorsphere formation in EGFR-positive cell lines. In addition, BBI608 efficiently inhibited G9a-exacerbated HER3 expression. Because HER3 facilitates EGFR-TKI resistance, our results indicate that the STAT3 blockade is a promising strategy that can be employed to eradicate EGFR-TKI-resistant lung cancers, including KRAS-mutant A549 and T790 M-mutant H1975 cells.

We previously demonstrated higher EGFR phosphorylation in HCC827-derived stem-like tumorspheres even without EGF treatment [18]; therefore, in the present model, the activation of STAT3 was considerable. Compared with HCC827 with an EGFR E746-A750 deletion, there was higher STAT3 phosphorylation and HER3 expression in H1975 cells (Fig. 3c), indicating that EGFR autophosphorylation led to the subsequent cascades. Previous studies have revealed that the activation of STAT3 is not only achieved by EGFR [17] but also by IL6-gp130 [34, 35]. Particularly, the inhibition of STAT3 phosphorylation increased the inhibitory effects of afatinib, an EGFR-TKI, in T790-mutant H1975 cells [34]. Therefore, targeting STAT3 is considered a potential strategy for overcoming EGFR-TKI resistance associated with KRAS and T790 M mutations in lung cancers. In addition, because we demonstrated that STAT3 induced HER3 expression in EGFR-positive lung cancers, the inhibition of STAT3 could prevent HER3-mediated EGFR-TKI resistance. Previous studies have indicated that the amplification of MET participates in EGFR-TKI resistance, which could be associated with MET-mediated HER3 expression [5] and MET-mediated AKT and MAPK signaling pathways [36]. A combined strategy simultaneously targeting STAT3 and MET could effectively prevent HER3-derived EGFR-TKI resistance.

In clinical trials, STAT3 inhibition has been reported to have remarkable therapeutic effects against various cancer types. For example, OPB-51822, a small-molecule STAT3 phosphorylation inhibitor targeting the SH2 domain (Tyr705/Ser727), exhibited high antitumor activity in EGFR-TKI-resistant NSCLC patients on a first-in-man phase I study [37]. In addition, STAT3-targeted RNAi therapies, such as AZD9150 (16-oligonucleotide antisense molecule targeting the 3′ untranslated part of STAT3), also exhibited single-agent antitumor activity in patients with lymphoma or NSCLC in a phase I dose escalation study [38]. However, the STAT3-HER3 cascade is a source of EGFR-TKI resistance. The amplification of oncogenes, such as HER2, also results in EGFR-TKI resistance [8]. In addition, previous studies have revealed that cancer stem cells that exhibit self-renewal and pluripotency are responsible for drug resistance and cancer recurrence [39]. The present study revealed that HER2 was overexpressed in HCC827-derived tumorspheres (Fig. 1d), and HER2 expression decreased following treatment with BBI608 (Fig. 3g) and A549shSTAT3 (Fig. 4e). Because there was no change in the expression of HER2 in A549shG9a (Fig. 4e), we concluded that STAT3 was a potential therapeutic target against lung cancers with EGFR-TKI resistance that was superior to G9a.

Increasing evidence suggests that microRNAs are involved in EGFR-mediated signaling pathways in lung cancers, including miR-145, which is downregulated and associated with TKI resistance targeting ERK, AKT, Oct4, c-MYC, EGFR, and NUDT1 [40]. The results indicate that miR-145 is a cell proliferation suppressor in lung adenocarcinoma by targeting EGFR and NUDT1 as an ERK and AKT phosphorylation inhibitor, which enhances gefitinib cytotoxicity in NSCLC [41, 42]. We further demonstrated that miR-145 was inhibited by G9a in the present study; thus, it played a major role in the regulation of HER3 expression. Because miR-145 has been reported to be a suppressor of HER3 translation in breast cancer [33], miR-145 downregulation by G9a could promote the overexpression of HER3 in lung cancers. In addition, we observed the downregulation of HER3 in A549shILF3 cells compared with A549shLuc cells, which validated that ILF3 enhanced HER3 expression [14]. A previous study reported that IL3 mediates miR-145 biogenesis and enhances the development of cancer stem cells in bladder cancer [43]. miR-145 targets EGFR [41], resulting in the downregulation of EGFR in A549ILF3 cells [14]. Therefore, YM155, an ILF3 inhibitor, caused a substantial decrease in EGFR [7], which led to the decrease in EGF-mediated G9a levels in the present study. Similarly, we confirmed that miR-200c, which is highly expressed and associated with epithelial–mesenchymal transition, invasion, and migration in NSCLC patients [44], was inhibited by G9a in A459 cells measured based on small RNAseq analyses, indicating that G9a not only regulated HER3 through the repression of miR-145-5p expression but also increased other oncogenes, such as the astrocyte leptin receptor, through the repression of miR-200c expression, which enhanced tumorigenesis in lung cancers.

## Conclusions

In conclusion, we demonstrated the STAT3-G9a-HER3 axis in lung cancer that evades EGFR-TKI therapies. The potential mechanism is through the repression of miR-145-5p expression, which specifically targets HER3 (Fig. 6). BBI608 and YM155 demonstrated similar effects, reducing the viability of lung cancer A549 cells and inhibiting STAT3-mediated G9a and HER3 expression. We propose that BBI608, which was selected from a panel kit, and YM155 are potential therapeutic agents against EGFR-positive lung cancers and may be combined with EGFR-TKIs for application in the eradication of lung cancers.

## Supplementary information

**Additional file 1: Table S1.** Differential genes in the HCC827-derived tumorspheres analyzed by RNAseq. (XLSX 1505 kb)

**Additional file 2: Table S2.** Differential genes in the BBI608-treated A549 cells analyzed by RNAseq. (XLSX 105 kb)

**Additional file 3: Table S3.** Differential genes in the YM155-treated A549 cells analyzed by RNAseq. (XLSX 160 kb)

**Additional file 4: Table S4.** Differential genes in the A549shSTAT3 cells analyzed by RNAseq. (XLSX 109 kb)

**Additional file 5: Table S5.** Differential genes in the A549shG9a cells analyzed by RNAseq. (XLSX 113 kb)

**Additional file 6: Table S6.** The decreased 309 genes overlapping between BBI608- and YM155-treated A549 cells, and decreased 245 genes overlapping between A549shSTAT3 and A549shG9a cells. (XLSX 15 kb)

**Additional file 7: Table S7.** Differential miRNAs in the A549shILF3 cells analyzed by small RNAseq. (XLSX 22 kb)

**Additional file 8: Table S8.** Differential miRNAs in the A549shG9a cells analyzed by small RNAseq. (XLSX 16 kb)

**Additional file 9: Figure S1.** BBI608 is a potential therapeutic agent against lung cancers. A panel kit containing 172 compounds was used to search for therapies effective against EGFR-positive HCC827, A549, H1975, and EGFR-negative H520 cell lines. The effective agents were selected based on a cell viability level lower than 40%. Among the therapies, only BBI608 markedly reduced cell viability against HCC827, A549, and H1975 rather than H520 cells. (TIF 2396 kb)

**Additional file 10: Figure S2.** Knockdown of G9a did not affect cell viability in A549 cells and in A549-derived tumor xenografts. (A) G9a was knockdowned using shRNA techniques, that did not reduce cell viability in A549 cells, and (B) in a A549-derived tumor xenograft model. NS, no significant. (TIF 145 kb)

**Additional file 11: Figure S3.** There were 55 reduction genes among BBI608 (BBI)-, YM155 (YM)-, shSTAT3, and shG9a-treated A549 cells (Additional file 6: Table S6), which were subsequently analyzed using NetworkAnalyst. (A) The 55 genes were classified using PANTHER (http://www.pantherdb.org/) based on molecular functions. The genes were listed based on their molecular functions, including binding (24 genes), catalytic activity (16 genes), molecular function regulator (5 genes), molecular transducer activity (3 genes), structural molecule activity (1 gene), transcription regulator activity (2 genes), and transporter activity. (B) NetworkAnalyst revealed that the ERBB signaling pathway was the major inhibitory pathway, particularly reducing *ERBB3* expression. (C) STAT3-G9a-regulated genes were compared with miR-145-5p-targeted genes from TargetScan resulted in four overlapping genes, including *PLA2G4A*, *MTUS1*, *ERBB3*, and *TSKU*. (TIF 912 kb)

**Additional file 12: Figure S4.** BBI608 and UNC0642 led to apoptosis in A549 cells. Apoptosis was detected in BBI608- and UNC0642-treated A549 cells using a flow cytometry, whereas FL1-H and FL2-H represented annexin-FITC and propidium iodide staining, respectively. Results indicate that BBI608 and UNC0642 caused A549 apoptosis from 1.06 to 45.08% by 1 μg/mL concentration and from 0.17 to 11.85% by 10 μg/mL concentration, respectively, for 4 h incubation. (TIF 396 kb)

## Data Availability

All data generated or analyzed during this study are included in this published article and its Additional files.

## Abbreviations

EGFR: Epidermal growth factor receptor
FBS: *Fetal bovine serum*
ILF3: Interleukin enhancer-binding factor 3
shRNA: Short-hairpin RNA
STAT3: Transducer and activator of transcription 3
TKIs: Tyrosine kinase inhibitors
*TPMs*: Transcripts per million clean tags
