# Supplementary figures and images for: STAT3 induces G9a to exacerbate HER3 expression for the survival of epidermal growth factor receptor-tyrosine kinase inhibitors in lung cancers
Source: BMC Cancer. 2019 Oct 16;19:959. doi: 10.1186/s12885-019-6217-9 (PMC6796430; doi:10.1186/s12885-019-6217-9)

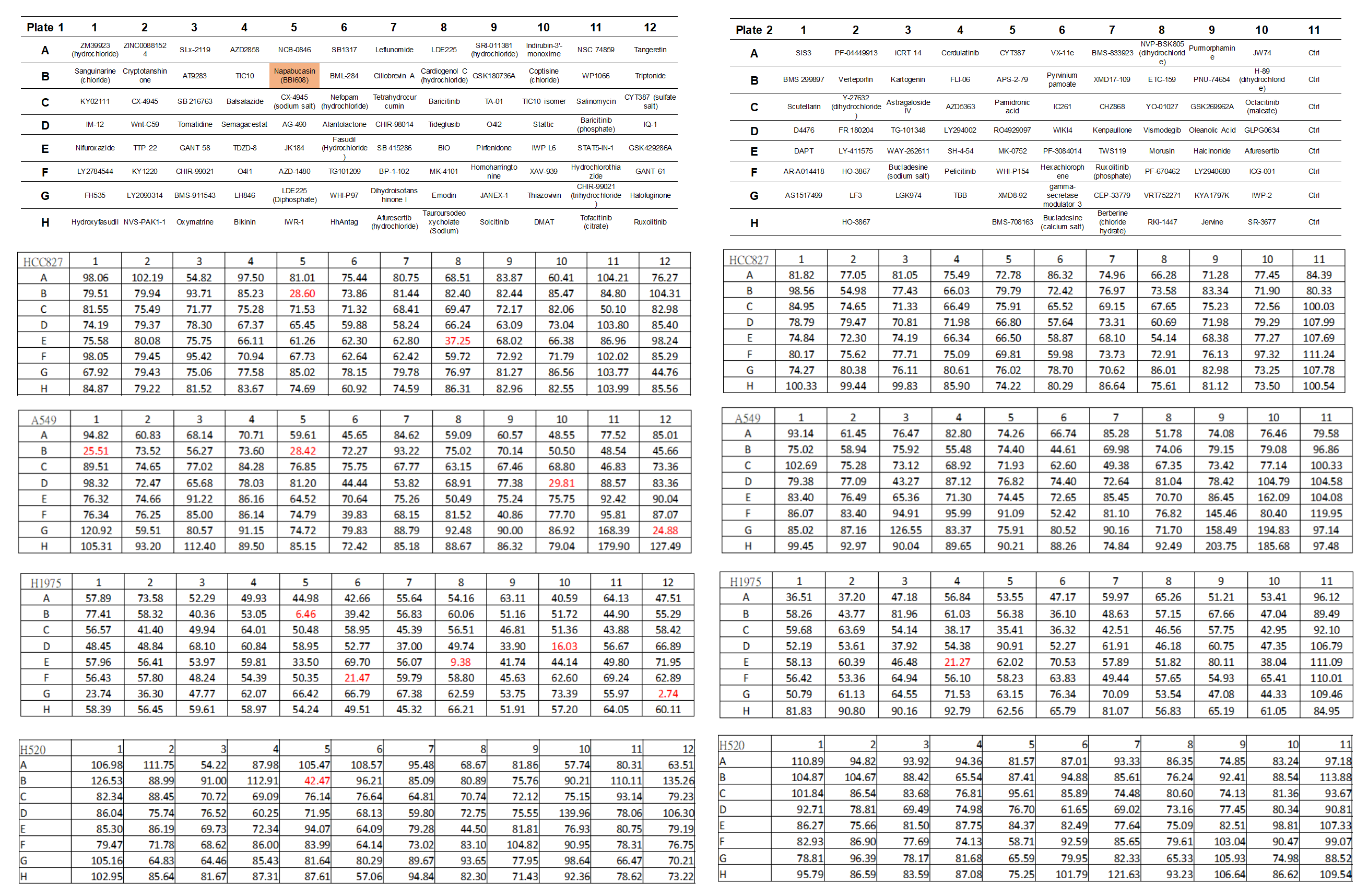

Supplement: Supplementary file 9 — Additional file 9: Figure S1. BBI608 is a potential therapeutic agent against lung cancers. A panel kit containing 172 compounds was used to search for therapies effective against EGFR-positive HCC827, A549, H1975, and EGFR-negative H520 cell lines. The effective agents were selected based on a cell viability level lower than 40%. Among the therapies, only BBI608 markedly reduced cell viability against HCC827, A549, and H1975 rather than H520 cells. (TIF 2396 kb) [file 12885_2019_6217_MOESM9_ESM.tif]

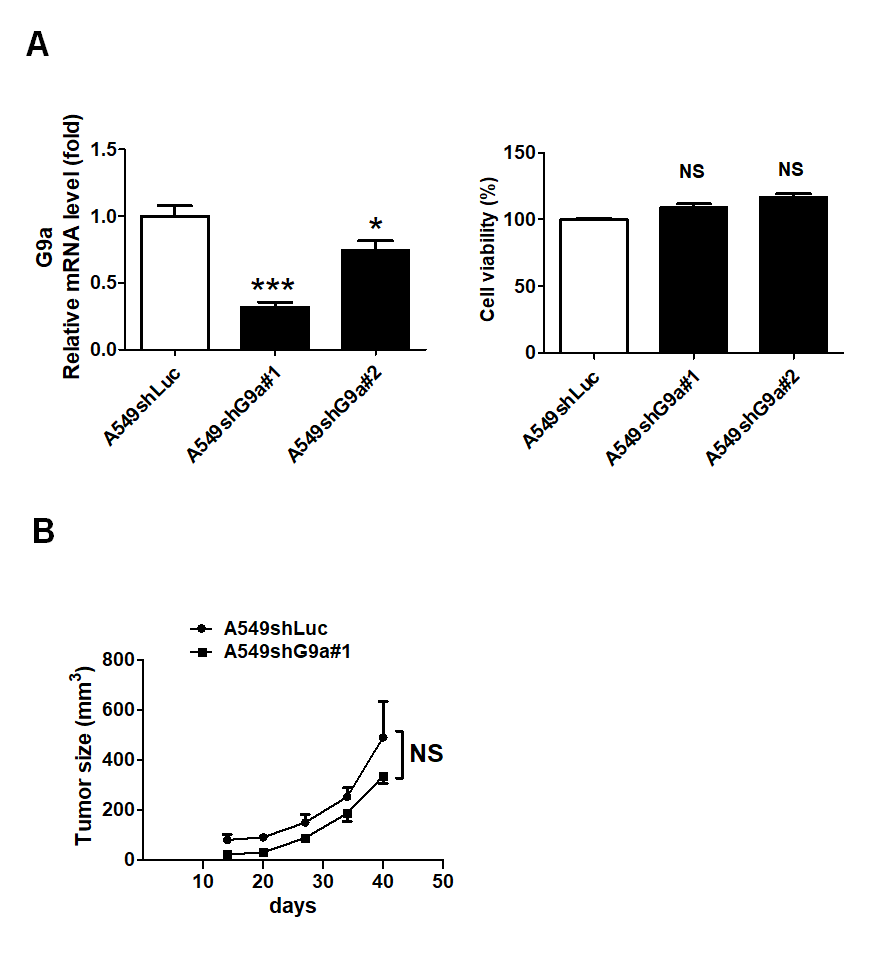

Supplement: Supplementary file 10 — Additional file 10: Figure S2. Knockdown of G9a did not affect cell viability in A549 cells and in A549-derived tumor xenografts. (A) G9a was knockdowned using shRNA techniques, that did not reduce cell viability in A549 cells, and (B) in a A549-derived tumor xenograft model. NS, no significant. (TIF 145 kb) [file 12885_2019_6217_MOESM10_ESM.tif]

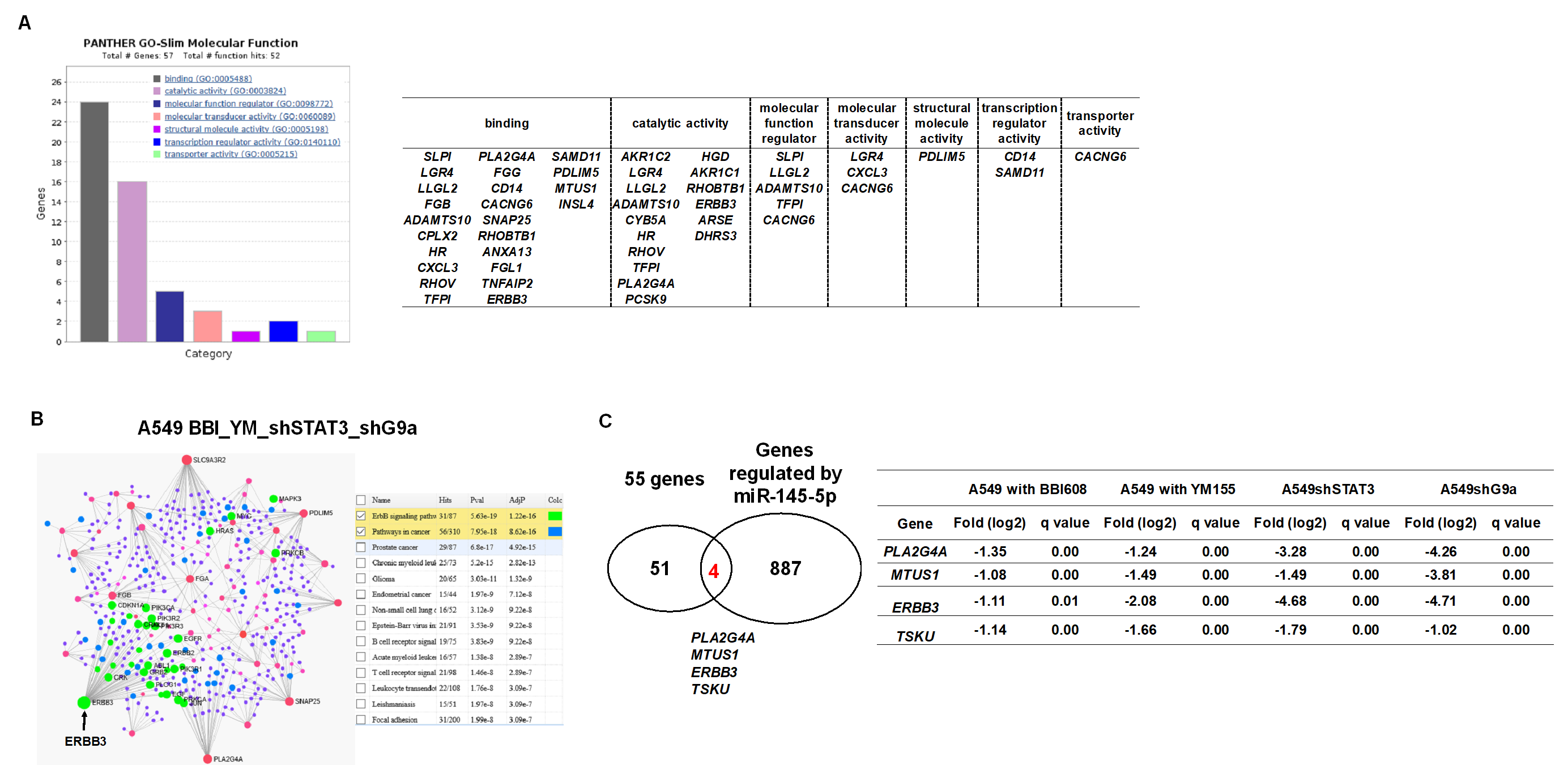

Supplement: Supplementary file 11 — Additional file 11: Figure S3. There were 55 reduction genes among BBI608 (BBI)-, YM155 (YM)-, shSTAT3, and shG9a-treated A549 cells (Additional file 6: Table S6), which were subsequently analyzed using NetworkAnalyst. (A) The 55 genes were classified using PANTHER (http://www.pantherdb.org/) based on molecular functions. The genes were listed based on their molecular functions, including binding (24 genes), catalytic activity (16 genes), molecular function regulator (5 genes), molecular transducer activity (3 genes), structural molecule activity (1 gene), transcription regulator activity (2 genes), and transporter activity. (B) NetworkAnalyst revealed that the ERBB signaling pathway was the major inhibitory pathway, particularly reducing ERBB3 expression. (C) STAT3-G9a-regulated genes were compared with miR-145-5p-targeted genes from TargetScan resulted in four overlapping genes, including PLA2G4A, MTUS1, ERBB3, and TSKU. (TIF 912 kb) [file 12885_2019_6217_MOESM11_ESM.tif]

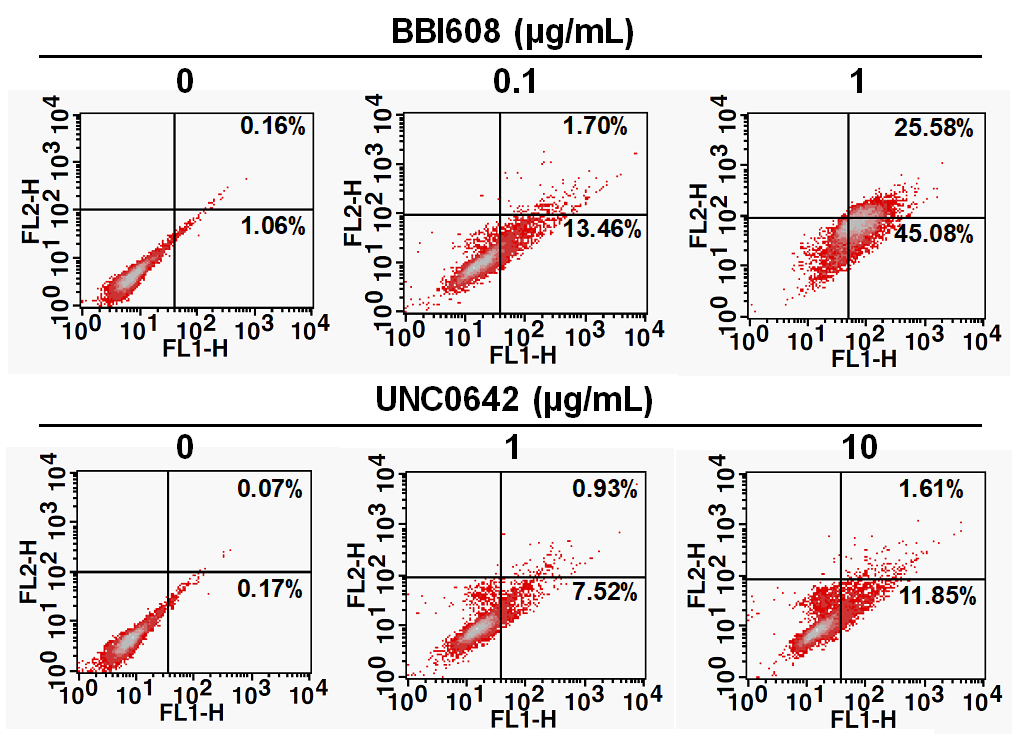

Supplement: Supplementary file 12 — Additional file 12: Figure S4. BBI608 and UNC0642 led to apoptosis in A549 cells. Apoptosis was detected in BBI608- and UNC0642-treated A549 cells using a flow cytometry, whereas FL1-H and FL2-H represented annexin-FITC and propidium iodide staining, respectively. Results indicate that BBI608 and UNC0642 caused A549 apoptosis from 1.06 to 45.08% by 1 μg/mL concentration and from 0.17 to 11.85% by 10 μg/mL concentration, respectively, for 4 h incubation. (TIF 396 kb) [file 12885_2019_6217_MOESM12_ESM.tif]
